# Assessing Historical Fish Community Composition Using Surveys, Historical Collection Data, and Species Distribution Models

**DOI:** 10.1371/journal.pone.0025145

**Published:** 2011-09-22

**Authors:** Ben Labay, Adam E. Cohen, Blake Sissel, Dean A. Hendrickson, F. Douglas Martin, Sahotra Sarkar

**Affiliations:** 1 Texas Natural History Collections, University of Texas, Austin, Texas, United States of America; 2 Section of Integrative Biology, University of Texas, Austin, Texas, United States of America; 3 Department of Philosophy, University of Texas, Austin, Texas, United States of America; Institute of Marine Research, Norway

## Abstract

Accurate establishment of baseline conditions is critical to successful management and habitat restoration. We demonstrate the ability to robustly estimate historical fish community composition and assess the current status of the urbanized Barton Creek watershed in central Texas, U.S.A. Fish species were surveyed in 2008 and the resulting data compared to three sources of fish occurrence information: (i) historical records from a museum specimen database and literature searches; (ii) a nearly identical survey conducted 15 years earlier; and (iii) a modeled historical community constructed with species distribution models (SDMs). This holistic approach, and especially the application of SDMs, allowed us to discover that the fish community in Barton Creek was more diverse than the historical data and survey methods alone indicated. Sixteen native species with high modeled probability of occurrence within the watershed were not found in the 2008 survey, seven of these were not found in either survey or in any of the historical collection records. Our approach allowed us to more rigorously establish the true baseline for the pre-development fish fauna and then to more accurately assess trends and develop hypotheses regarding factors driving current fish community composition to better inform management decisions and future restoration efforts. Smaller, urbanized freshwater systems, like Barton Creek, typically have a relatively poor historical biodiversity inventory coupled with long histories of alteration, and thus there is a propensity for land managers and researchers to apply inaccurate baseline standards. Our methods provide a way around that limitation by using SDMs derived from larger and richer biodiversity databases of a broader geographic scope. Broadly applied, we propose that this technique has potential to overcome limitations of popular bioassessment metrics (e.g., IBI) to become a versatile and robust management tool for determining status of freshwater biotic communities.

## Introduction

A reference condition is critical to the interpretation of bioassessment data and indicators of ecosystem health. Ideally, benchmarks defining a biotic reference condition are determined from sites undisturbed by anthropogenic stressors, thus representing continuity with a historical condition and community composition [1]. However, since pristine habitats and even minimally disturbed sites are increasingly rare or non-existent in many regions, managers often rely on least-disturbed sites to determine benchmarks [2]–[4]. As reference sites continue to experience anthropogenic stressors, without strong historical reference data, the human-perceived baselines are prone to shift. The term “shifting baseline” was developed in the marine fisheries literature [5] to refer to what amounts to a pronounced tendency over time, and especially over human generations, for true historical conditions to be forgotten, distorted or overlooked. Over time, shifting baselines result in management for steadily decreasing biodiversity or habitat quality. This process undermines our attempts to manage for sustainability into the future as managers rely on incomplete perspectives as to what a “natural” assemblage is for a given area and what factors shape it. This is especially acute in freshwater systems where historical data are relatively sparse compared with marine fisheries where shifting baselines were first documented, and where the dendritic nature of streams and rivers serve to aggregate stressors over large spatial and temporal scales, resulting in widespread effects [6]–[8] and difficulty in attributing causal mechanisms [9], [10]. Modeling and historical reconstruction of community compositions prior to human alteration could help managers correctly set and maintain baselines.

While it is generally difficult to estimate historical benchmarks, most often due to insufficient pre-imperilment data for a particular study area, this does not justify accepting (e.g. [11]) that obtaining or improving a historical perspective of biotas will never be possible. Advances in information technology and worldwide efforts to compile, digitize, and make biodiversity data available (e.g., NatureServe [www.natureserve.org], Global Biodiversity Information Facility [www.gbif.org]) have recently improved our perception of the diverse scales of anthropogenic alteration of the environment. Simultaneously, development of new tools and techniques help summarize and utilize these biodiversity datasets to aid in historical reconstruction of biotic communities. Species distribution modeling (SDM) is one such tool that is increasingly used in many disciplines, including applied fields of systematic conservation planning [12]–[14], climate change studies [15], [16], disease ecology [17]–[20], and invasive species research [21], [22], and it can be applied in ways that allow us to use regional biotic data to reconstruct historical biotas for areas lacking actual historical occurrence records. This technique converts disparate occurrence records into continuous probabilities of occurrence that predict habitat suitability. SDMs are therefore more amenable to diverse mathematical analyses as performed in geographical information systems than are the raw occurrence data [23], which typically lack proper temporal and spatial representation for direct use in most comparative or trend analyses commonly used for assessing changes in biological communities. Through the incorporation of these disparate and temporally diverse historical occurrence data with environmental variables accounting for only broad-scale physiological and biogeographical constraints, we propose that SDMs used as described in this study provide a more robust and quantifiable estimation of historical habitat suitability than bioassessment techniques using variable quality reference conditions [24], [25]. This paper explores this premise, and provides a novel integration of SDMs for historical community reconstruction and application to stream bioassessment. By producing SDMs for all taxa in a potential pool of species, we approximate historical community composition across a small watershed with sparse observational data.

The analysis presented here uses surveys, historical species occurrence data, and SDMs to reconstruct a watershed's historical fish community composition and relate it to its current composition. The study area, Barton Creek watershed, Texas, is especially relevant for this analysis as it is an urban/suburban watershed with a long history of hydrologic alteration, heavy urban development pressure, and it has historical fish collection records adequate for relatively robust study of changes over time. Our objectives were to: (i) compile historical data for this watershed's fish community, (ii) use regional and study area-specific occurrence data to create SDMs that allow quantitative inference of the historical fish community, and (iii) assess the current biotic condition of the study area by comparing a recent (2008) assemblage survey to baselines provided by the historical data, a nearly identical survey conducted 15 years earlier, and the SDM-derived modeled community. Each of these diverse data sources restrict or limit interpretation in unique ways. Contemporary surveys offer only a brief snapshot, historical collection data are temporally, spatially, and methodologically disparate, and a modeled reconstruction is only an approximation of reality. Analyzed together, however, the separate limitations of these data sources are largely overcome to provide a highly informative and useful perspective over expanded temporal and spatial scales, and allow for better understanding of historical community compositions and how the contemporary community compares.

## Methods

### Study Area

**Figure 1:**
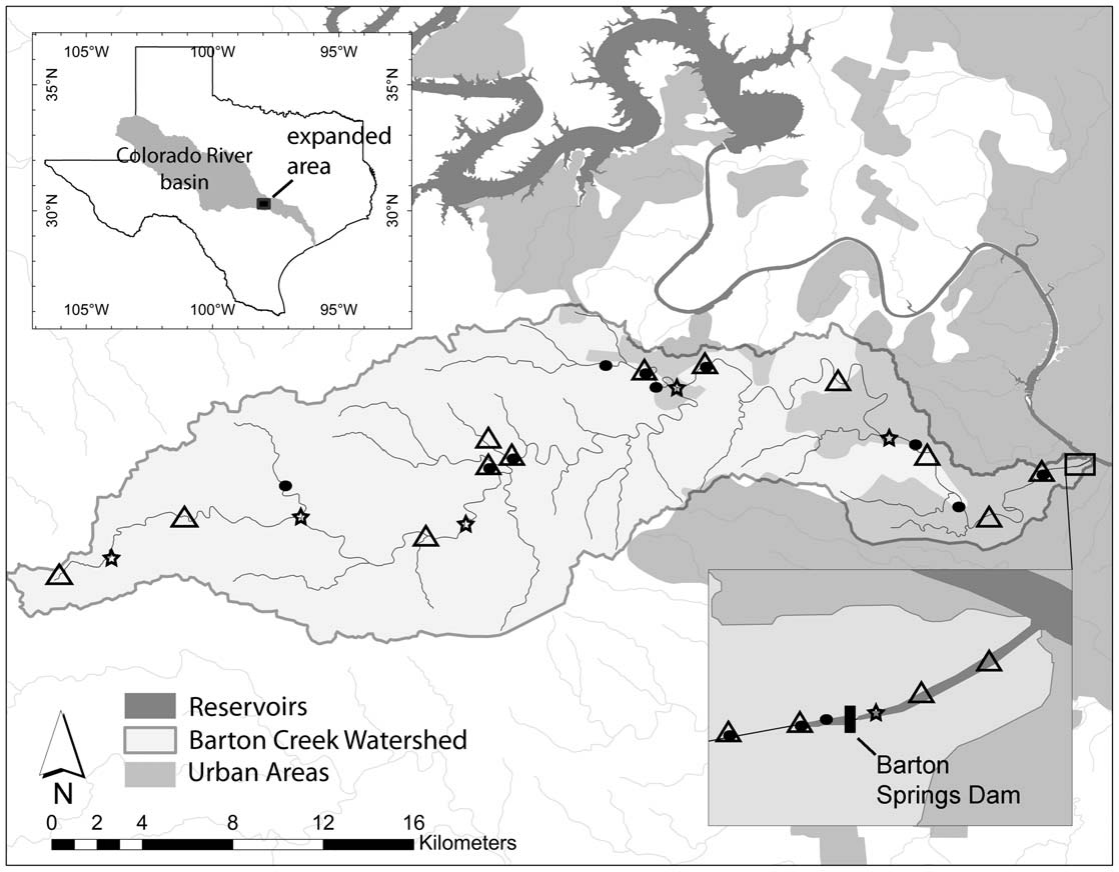
Map of Barton Creek watershed illustrating survey collection localities, location of Barton Springs dam, and relationship of the watershed to the larger Colorado River basin. Black circles represent locations sampled during the 2008 survey, stars represent the six seasonally sampled sites common to both the 1993 and 2008 surveys, and triangles represent 1993 survey and historical collections.

The study area was the Barton Creek Watershed (BCW), a 281 km^2^ drainage that empties into Ladybird Lake, a power-plant supply and flood control reservoir on the Colorado River, in downtown Austin, Texas, USA (Figure 1). BCW contains predominantly intermittent streams with ephemeral tributaries, but throughout the watershed are perennial reaches maintained by approximately 60 springs, small dams, and natural plunge pools. The lowest two km of Barton Creek passes through an aquifer resurgence reach where numerous springs, including Texas' fourth largest (Barton Springs), bring water to the surface. These springs provide habitat for the endangered Barton Springs Salamander (*Eurycea sosorum*) and the federal candidate for listing, Austin Blind Salamander (*Eurycea waterlooensis*) [26]. Elevations in the watershed range from approximately 400m above sea level near the headwaters to 130 m at the mouth. Area annual rainfall is approximately 81 cm. The furthest upstream and downstream United States Geological Survey gaging stations record average annual discharges (for 1979–2010) of 0.45(0.43sd) and 0.64 (0.23sd) cubic meters per second, respectively.

BCW and surrounding drainages have experienced a long history of hydrologic alteration. The reservoir that BCW drains into, Ladybird Lake, was created in 1960 with construction of Longhorn Dam, that maintains a nearly constant-level impoundment that inundates the lower 1 km of Barton Creek (Figure 1). Longhorn Dam, approximately 5.5 km downstream from the mouth of Barton Creek, is the most downstream of seven large dams on the Colorado River within central Texas that create a series of flood control and hydroelectric reservoirs known as the Highland Lakes. All but Longhorn Dam were constructed before 1951. Below Longhorn Dam the river flows freely for 542 km as it drops 130m to the Gulf of Mexico. Independent of extensive water management alterations on the Colorado River, Barton Creek has also experienced a long history of biotic and hydrologic alteration. In 1881, Texas Parks and Wildlife developed the state's first fish hatchery at Barton Springs, propagating the non-native Common Carp, *Cyprinus carpio* [27]. In 1929 the City of Austin dammed Barton Creek at Barton Springs to create a recreation destination that is still in operation. Furthermore, a recent study projects that future groundwater extraction rates will result in significant declines in spring discharges within the watershed and even a cessation of discharge for Barton Springs in severe drought conditions [28].

### Collection Data

Two comprehensive fish surveys were conducted in BCW, one from February through December 1993 and the second from April 2008 to February 2009 (hereafter referred to as the 1993 and 2008 surveys, respectively). Data from the 1993 survey were obtained from the survey's specimens and field notes deposited at the Texas Natural History Collection (TNHC), University of Texas at Austin, TX, USA. To the extent we could reconstruct the design based on field notes, the 2008 survey served as a replicate of the 1993 survey. Altogether, the surveys account for 37 (1993) and 34 (2008) collection events (collection event defined as collection of ≥one specimen from one site on one date) within one calendar year. Both surveys sampled the same six BCW mainstem sites (Figure 1) quarterly for one year, with the exception of two sites in 2008 that were sampled only twice as they were dry in two of the sampling quarters. The sampling protocol at the six mainstem sites in both surveys included sampling all available habitat using seines (0.48 cm and 0.64 cm mesh [stretch]) for 1–1.5 hours per site. Additionally, in an attempt to maximize sampling coverage and the probability of capturing all taxa, 13 (1993) and 14 (2008) sites, here referred to as supplementary, were scattered throughout the watershed and sampled once in each survey. The 1993 supplemental site sampling protocol is unknown; however, the spatial coverage and associated specimens housed at TNHC lead us to conclude that the 1993 survey provided a thorough assessment of the watershed's fish fauna. The 2008 supplemental site sampling protocol varied among sites depending on habitat availability. To maximize the probability of collecting all species present, collections were conducted during both night and day across all habitats available using diverse collecting gears: seines (0.48 cm and 0.64 cm mesh [stretch]), backpack electroshocker, submersed and floating funnel minnow traps, multi-filament gill nets (various mesh sizes between 35 mm and 95 mm [stretch]), trammel nets, hoop nets, trot lines, and frame nets. For both surveys, all individuals collected were identified in the field, counted and released, except for vouchers from each collection event that were anesthetized, preserved in 10% formalin and taken to the laboratory for positive identification and deposition at TNHC.

Data for historical fish collections from Barton Creek were obtained from the recently compiled Fishes of Texas (FoTX) database [29], [30] maintained by TNHC. This high quality, comprehensive online database (http://www.fishesoftexas.org-in Beta as of February 25, 2011) is a compilation of records compiled from 40 institutions worldwide that contains over 80,000 museum-vouchered (specimen-based) occurrence records precisely georeferenced using standard protocols [31] and estimated to represent 95% of all specimen-vouchered fish collections ever made in the state of Texas. In addition to museum records, we compiled non-vouchered fish records by extracting them from TNHC-archived field notes and searches of both academic and gray literature. Only vouchered museum occurrence records were used in modeling (described below), while unvouchered data served as anecdotal historical occurrences used in comparative analysis and discussion.

### Species Distribution Modeling

#### Spatial Extent

The extent used for modeling was the political boundary of the state of Texas divided into a grid of 931,808 cells at a resolution of 30 arc-seconds. The average cell area was 0.73 km^2^.

#### Occurrence Records

Species distribution models were constructed for all fish species that are recorded in the FoTX database as occurring in the Colorado River basin (see Colorado River basin within Texas in the inset of Figure 1) and that are listed by Hubbs et al. [32] as being freshwater or freshwater-estuarine, excluding marine and strictly estuarine species. We assumed that these represent the entire potential species pool for BCW. Non-native species were included to provide managers estimates of potential habitat suitability for these taxa, not to give insights into historical conditions. Records from the 2008 survey were excluded from model development so that they could be independently compared to the models. Records with > one km potential georeferencing error (radius) were also excluded to assure input occurrences closely corresponded in spatial resolution to environmental layers used in modeling (see Variable Set Selection below). This spatial error threshold of one km approximately matches the grid cell resolution of 30 arc-seconds (which approximates one km at the Equator), but is slightly larger than the longitudinal boundary of the average cell size (0.73 km^2^) due to geographic projection at the latitude of Texas. However, the maximum entropy algorithm used for analysis (see Model Construction below) has been shown not to be affected by spatial errors in occurrence datasets with standard deviations up to five km [33], [34]. Occurrence records before 1950 were similarly excluded so that occurrence data were temporally congruent with climatic variables used (see Variable Set Selection below). Finally, since model performance stabilizes with respect to accuracy of prediction at about 10 records when using the maximum entropy model construction algorithm [35], [36], models were produced only for those species for which we had a minimum of 10 occurrences corresponding to at least 10 unique cells on the environmental layer grids.

#### Model Construction

Models were constructed using the maximum entropy algorithm encoded in the Maxent software package (Version 3.3.4; [37]), known to be robust for species distribution modeling with presence-only records [35], [36]. Maxent was parameterized following published recommendations [35], with models replicated 100 times withholding randomly in each replicate 40% of localities as ‘test’ records, with the remaining 60% serving as model ‘training’ records. Model performance was evaluated using a (threshold-independent) receiver operating characteristic (ROC) analysis and 11 internal binomial analyses of ‘training’ and ‘test’ occurrence omission. The ROC analysis characterizes model performance at all possible thresholds using the area under the curve (AUC), a measure of model performance independent of any threshold [38]. An optimal model with perfect discrimination would have an AUC of 1 while a model that predicted species occurrences at random would have an AUC of 0.5 [38].

#### Variable Set Selection

The environmental variable set used in the final models was selected by Texas fish experts at TNHC (AEC, DAH, BL, DFM) who chose the best model for each of a trial set of 16 species produced by 10 combinations of environmental variables and spatial extent (see the protocol described below). Expert opinion was used because preliminary model predictions differed greatly in spatial configuration depending on the number and type of such variables used to construct a model while internal model quality tests (AUC and binomial tests of omission) were consistent among them. Inclusion of expert opinion to identify biologically plausible models [39], [40] addresses criticism of standard internal tests to validate SDMs [41], [42], and increases the stringency of criteria used to validate model quality and helps prevent overfitting and variable dredging [43].

The following summarizes our process of model selection via expert opinion:

1. Sixteen fish species (Table 1) were chosen by Texas fish experts at TNHC on the basis of lacking substantial range extension outside of Texas and having high probability of capture using standard fish collection techniques. Thus these species were likely to have well-known distributions so that model performance could be verified via expert opinion.
2. The 10 SDMs were constructed for these 16 species by using subsets of the composite variable set and, for four variable sets, using two different extents of the study area (see Table 2 for full list of environmental variables and Table 3 for variable sets). The two extents include the political boundary of Texas, as this was the extent of both the species occurrence data and 10 supplemental variables, and an expanded extent of Texas that included all WWF-defined freshwater ecoregions (available at http://www.feow.org/index.php) that overlap with Texas.
3. All ten models for each of the 16 species were assigned random numbers and presented to the experts without them knowing the variables and extents used to create them. Models created using the expanded extent were clipped to the political extent for presentation to the experts.
4. Experts independently ranked models from one to five on the basis of accuracy of the models' depiction of the species distribution as they knew them, with one being poor and five being excellent. Independent rankings from each expert were averaged for analysis.
5. Due to the inherent variability in expert opinion analyses, subsequent model construction used the two highest-ranked variable sets, and for each species the model with the highest AUC was selected for incorporation into the modeled community construction. Table 4 shows which variable set (as numbered in Table 3) was used for each species.

**Table 1:** Species used in expert opinion analysis.

Genus species	Common name
*Cyprinella lepida*	Plateau Shiner
*Cyprinella proserpina*	Proserpine Shiner
*Cyprinodon rubrofluviatilis*	Red River Pupfish
*Dionda argentosa*	Manantial Roundnose Minnow
*Dionda diaboli*	Devils River Minnow
*Dionda nigrotaeniata*	Guadalupe Roundnose Minnow
*Dionda serena*	Nueces Roundnose Minnow
*Etheostoma lepidum*	Greenthroat Dater
*Macrhybopsis marconis*	Burrhead Chub
*Micropterus treculii*	Guadalupe Bass
*Moxostoma congestum*	Gray Redhorse
*Notropis amabilis*	Texas Shiner
*Notropis buccula*	Smalleye Shiner
*Notropis oxyrhynchus*	Sharpnose Shiner
*Percina apristis*	Guadalupe Darter
*Percina carbonaria*	Texas Logperch

**Table 2:** Environmental variables used in models.

Variable category	Description	Data Source
Topological	aspect	derived from altitude
	slope	derived from altitude
	compound topological index	derived from altitude
	altitude	Worldclim
Climate	**annual mean temperature**	Worldclim
	**mean diurnal range**	Worldclim
	isothermality	Worldclim
	temperature seasonality	Worldclim
	**max temperature of warmest month**	Worldclim
	**min temperature of coldest month**	Worldclim
	temperature annual range	Worldclim
	annual precipitation	Worldclim
	**precipitation of wettest month**	Worldclim
	**precipitation of driest month**	Worldclim
	**precipitation seasonality**	Worldclim
	precipitation of wettest quarter	Worldclim
	precipitation of driest quarter	Worldclim
	precipitation of warmest quarter	Worldclim
	precipitation of coldest quarter	Worldclim
Supplemental	karst regions	National Atlas
	natural regions	Texas Parks and Wildlife
	vegetation types	Texas Parks and Wildlife
	**freshwater ecoregions**	World Wildlife Foundation
	**terrestrial ecoregions**	World Wildlife Foundation
	potential evapotrasporation (avg. over 8-digit HUC)	Center for Research in Water Resources, UT Austin
	major aquifers	Texas Water Development Board
	minor aquifers	Texas Water Development Board
	major river basins	Texas Parks and Wildlife
	8-digit hydrologic unit code (HUC)	U.S.G.S. - National Hydrography Dataset
	**streams and rivers**	U.S.G.S. - National Hydrography Dataset
	stream order	U.S.G.S. - National Hydrography Dataset
	12-digit HUCs containing springs	U.S.G.S. - Database of Historically Documented Springs

The reduced set of 7 climatic layers that resulted in higher AUC values for certain species (see methods) is marked in bold. Supplemental layers in bold are those expandable into Mexico used when modeling with the expanded extent for the model selection process (see methods).

**Table 3:** Variable sets used in variable set selection process.

Set	Variables	Extent
1	15 bioclim, 4 topo	political boundary
2	15 bioclim, 4 topo	expanded boundary
**3**	**15 bioclim, 4 topo, 13 supplemental**	**political boundary**
4	15 bioclim, 4 topo, 3 extendable supp.	political boundary
5	15 bioclim, 4 topo, 3 extendable supp.	expanded boundary
6	7 bioclim, 4 topo	political boundary
7	7 bioclim, 4 topo	expanded boundary
8	7 bioclim, 4 topo, 3 extendable supp.	political boundary
**9**	**7 bioclim, 4 topo, 13 supplemental**	**political boundary**
10	7 bioclim, 4 topo, 3 extendable supp.	expanded boundary

Variable sets 3 and 9 (in bold) were selected by the expert opinion process as those producing the most reliable distributions for the 16 species with ‘known ranges’ (see methods).

**Table 4:** Fishes known from the Colorado River basin successfully modeled and accepted under study criteria (see text).

Genus species	Max BCW Prob.	1993	2008	Historical	Total	Avg. test AUC	Variable set^+^	Model records
*Gambusia affinis*	0.97	29	31	18	78	0.9285	3	750
*Lepomis megalotis*	0.97	21	27	10	58	0.9447	3	601
*Poecilia latipinna^I^*	0.97	-	-	-	0	0.9755	3	69
*Campostoma anomalum*	0.96	25	25	11	61	0.9776	3	208
*Lepomis auritus* ***^I^***	0.96	30	32	11	73	0.9718	3	140
*Notropis texanus*	0.96	7	2	5	14	0.9840	3	168
*Fundulus notatus*	0.95	1	-	5	6	0.9643	3	224
*Lepomis macrochirus*	0.95	27	31	8	66	0.9369	3	537
*Micropterus salmoides*	0.95	22	26	8	56	0.9401	3	368
*Micropterus treculii*	0.95	13	5	5	23	0.9833	3	86
*Cyprinella venusta*	0.94	32	30	12	74	0.9647	3	487
*Ictalurus punctatus*	0.94	1	15	3	19	0.9455	3	259
*Lepomis microlophus*	0.94	2	8	3	13	0.9521	3	143
*Lepomis miniatus*	0.93	6	10	6	22	0.9700	3	142
*Ameiurus natalis*	0.92	1	4	2	7	0.9375	3	126
*Herichthys cyanoguttatum* ***^I^***	0.92	-	24	4	28	0.9795	3	145
*Pimephales vigilax*	0.92	-	-	1	1	0.9505	3	464
*Etheostoma lepidum*	0.91	9	11	8	28	0.9859	3	68
*Etheostoma spectabile*	0.90	-	-	2	2	0.9834	3	120
*Cyprinella lutrensis*	0.89	2	1	1	4	0.9312	3	564
*Lepomis gulosus*	0.89	1	1	-	2	0.9450	3	232
*Astyanax mexicanus* ***^I^***	0.88	7	5	10	22	0.9791	3	133
*Micropterus punctulatus*	0.88	4	14	3	21	0.9748	3	167
*Notropis volucellus*	0.88	3	7	-	10	0.9743	3	206
***Opsopoeodus emiliae***	**0.87**	-	-	-	**0**	0.9745	3	195
*Percina sciera*	0.87	-	-	1	1	0.9787	3	194
***Notropis shumardi***	**0.81**	-	-	-	**0**	0.9742	3	32
*Pomoxis annularis*	0.80	1	-	-	1	0.9539	3	132
***Lepomis humilis***	**0.79**	-	-	-	**0**	0.9463	3	120
*Dorosoma cepedianum*	0.78	-	-	1	1	0.9414	3	185
*Moxostoma congestum*	0.77	3	9	2	14	0.9821	3	81
*Carpiodes carpio*	0.68	-	-	1	1	0.9448	3	122
***Hybopsis amnis***	**0.63**	-	-	-	**0**	0.9887	9	71
***Percina macrolepida***	**0.63**	-	-	-	**0**	0.9525	3	69
*Gambusia geiseri^I^*	0.59	-	-	-	0	0.9864	9	31
*Notropis amabilis*	0.57	-	-	2	2	0.9919	3	111
*Dionda nigrotaeniata*	0.56	-	-	1	1	0.9893	3	31
***Notropis stramineus***	**0.54**	-	-	-	**0**	0.9746	3	62
***Notropis buchanani***	**0.52**	-	-	-	**0**	0.9650	3	92
*Noturus gyrinus*	0.42	-	-	-	0	0.9649	3	102
*Etheostoma chlorosoma*	0.34	-	-	-	0	0.9834	3	182
*Fundulus zebrinus*	0.31	-	-	-	0	0.9575	3	101
*Lythrurus fumeus*	0.31	-	-	-	0	0.9838	3	179
*Percina carbonaria*	0.30	-	1	-	1	0.9859	3	35
*Phenacobius mirabilis*	0.29	-	-	-	0	0.9581	9	40
*Cyprinodon variegatus*	0.23	-	-	-	0	0.9560	3	34
*Lepisosteus oculatus*	0.17	-	-	-	0	0.9612	3	43
Macrhybopsis marconis*	0.15	-	-	1*	1*	0.9963	9	35
*Hybognathus placitus*	0.13	-	-	-	0	0.9691	9	43
*Aphredoderus sayanus*	0.06	-	-	-	0	0.9794	3	114
*Minytrema melanops*	0.05	-	-	-	0	0.9792	3	71
*Etheostoma gracile*	0.03	-	-	-	0	0.9834	3	142
*Notropis oxyrhynchus*	0.03	-	-	-	0	0.9811	3	30
*Macrhybopsis hyostoma*	0.02	-	-	-	0	0.9840	3	65
*Pomoxis nigromaculatus*	0.01	-	-	-	0	0.9644	3	51
*Etheostoma proeliare*	0	-	-	-	0	0.9935	3	69
*Cyprinodon rubrofluviatilis*	0	-	-	-	0	0.9834	3	47

Maximum probability of occurrence in BCW, number of collecting events documenting species occurrences in BCW from surveys and historical collections, average test AUC, variable set used in model construction, and number of records used in training and testing models shown for each species. Bold marks native species with >0.5 max watershed probability, but lacking documentation by collections from the BCW. (*) represents a vouchered record of “*Macrhybopsis sp*,” here arbitrarily identified as *M. marconis*, but that could be *M. hyostoma*. (***^I^)*** represents species introduced (not native) to the Colorado River basin. (**^+^)** Refer to Table 3 for details on variable sets.

The full variable set consists of four topographical variables (elevation, slope, aspect, and composite topographical index), 15 bioclimatic variables, and a supplemental set of 13 categorical variables depicting various hydrologic, geologic, and biotic geographies thought to correlate with fish distributions (Table 2). The bioclimatic variables were obtained from the WorldClim database (www.worldclim.org), which contains global climate layers averaged from 1950–2000. Four of the WorldClim bioclimatic variables (mean temperatures of the wettest quarter, driest quarter, warmest quarter and coldest quarter) were excluded because of known artifactual discontinuities in Texas [17].

Subsets of environmental variables were compared to assess whether added parameters caused by more variables, especially categorical variables, resulted in overfitting due to over-parameterization [44] or if they were necessary to obtain high quality SDMs. The comparisons of extents were carried out because (i) using the political extent of Texas, although an arbitrary boundary ignored by fish, permitted the use of biologically meaningful environmental variables that were restricted to this extent, (ii) the political extent corresponded spatially to the occurrence data so the expanded extent could have produced overfitting in models of species that occur only or primarily on the edge of Texas [35], [45], [46], and (iii) the political extent could have produced models with ‘truncated response curves’ for species located near the edge of Texas [35], [45], [47].

#### Data Analysis

To test for overall differences in the watershed assemblage between 1993 and 2008, a Mantel Test (Zt software; http://bioinformatics.psb.ugent.be/webtools/zt/) was performed on data matrices of Bray-Curtis similarity distances for species abundance data of the surveys' six mainstem sites. We present trends in abundance for select native and non-native species to provide a perspective on recent regime shifts of BCW's fish community based on survey results.

Species models developed as described above were considered reliable and retained for modeled community construction if they had: (i) average AUC over 100 replicates >0.9, (ii) a *p*-value <0.05 for all internal training and test binomial occurrence omission analyses among all replicates performed by Maxent, and (iii) a less than five percent difference between average test and training AUC. Despite the maximum entropy algorithm's design to take into account correlations between variables, using a large number of variables raises dangers of over-fitting, thus this third criterion, as well as the variable set-selection process as described above, conservatively eliminate the risk of models showing signs of over-fitting [17], [44].

From each species' model meeting all of the above criteria, maximum modeled probability of occurrence in BCW was extracted to serve as a coarse-scale-proxy for the potential of establishment within BCW. The models generally account for species-specific physiological constraints as determinants of distributions by virtue of being highly correlated with the continuous environmental variables used (climatic and topographic sets) [48], [49]. Furthermore, historical zoogeographic barriers to dispersal (e.g. drainage divides) are taken into account by inclusion of categorical variables (supplemental variable set) in the models and in only considering those species known to occur within the Colorado basin. Therefore, a high predicted probability of presence within the watershed when the collection data indicate absence is strong support for the hypothesis that a species formerly occurred in BCW and that its absence could be the product of factors related to human development or biotic interactions.

## Results

### Collection Data

The 1993 survey yielded a total of 12,726 individuals representing 28 species in nine families and at the six mainstem sites, 7,634 individuals from 21 species (eight families) were collected. The 2008 survey yielded a total of 11,779 individuals representing 26 species in eight families and at the six mainstem sites 7,081 individuals from 23 species (eight families) were collected. In both surveys, maximum species richness (16) was recorded at Barton Creek below Barton Springs dam, while the lowest species richness (four) was recorded at the most-upstream site. In both surveys, these two sites have greater difference in species composition than was found among any of the four mid-reach sites, a finding that is largely attributable to low species richness at the headwater sites (presumably subject to stochastic extirpation and colonization) and high species richness at the downstream site, which is influenced by mainstem (river/Ladybird reservoir) vagrants.

Mantel test comparisons did not detect significant differences in overall watershed-wide faunal assemblages between the 1993 and 2008 surveys (*r* = 0.69; *p* = 0.075). This overall similarity is not surprising since above Barton Springs dam, where the majority of our samples were taken, 21 of the 25 total species recorded were shared between the 1993 and 2008 surveys. *Lepomis macrochirus*, *Micropterus salmoides*, *Cyprinella venusta*, *Gambusia affinis*, *Lepomis auritus*, *Lepomis megalotis*, and *Campostoma anomalum* were the most widespread and *C. venusta*, *G. affinis*, *C. anomalum*, *L. macrochirus* and *L. auritus* were the most abundant species. However, closer inspection of the data reveals notable differences between surveys. The non-native *Herichthys cyanoguttatum*, overall the sixth most abundant species in 2008, was not collected in 1993. Another non-native, *L. auritus*, increased at the six quarterly sampled mainstem sites from 3.5% of total catch in 1993 to 18% in 2008. Differences between surveys varied geographically. Below Barton Springs dam, where the creek is heavily affected by the reservoir, 11 of the 22 recorded species were shared between surveys. At this site *Fundulus notatus*, *C. anomalum*, *Menidia beryllina*, *Notemigonus crysoleucas*, and *Notropis texanus* were not collected in 2008 but were found in 1993. Conversely, absent at this same site in 1993 but present in 2008 were *Ameiurus natalis, Cyp. carpio, H. cyanoguttatum, L. megalotis, Lepomis microlophus,* and *Percina carbonaria*. Also of special interest was that the native keystone herbivore [31], *C. anomalum,* comprised 32% of the total catch below Barton Springs dam in 1993, being found in all four seasonal collections, but was not collected there in any of the 2008 collections. Similarly, upstream of Barton Springs dam *C. anomalum* abundances declined from 15% of the total catch in 1993 to 6% in 2008.

TNHC's Fishes of Texas database documented 24 historical collecting events in BCW. These collections made by numerous collectors at ten locations (Figure 1) on 22 dates and using generally unspecified methods produced 134 species occurrence records documenting 41 taxa in 13 families. *G. affinis* is represented in 53% of historical collecting events, followed by *C. venusta* (35%), *C. anomalum* and *L. auritus* (both 32%), and *A. mexicanus* and *L. megalo*tis (both 29%). The oldest museum-vouchered records found were Jordan's and Gilbert's 1884 collection of the lower one km of the creek, Barton Springs downstream to the mouth at the Colorado River [50]. This pre-dam collection documented 24 species, of which seven (*Aplodinotus grunniens*, *Carpiodes carpio*, *Ictiobus* sp., *Macrhybopsis sp*., *Notropis amabilis*, *Notropis atherinoides*, and *Pimephales vigilax*) were never again documented to occur in BCW. The *N. atherinoides* record (USNM 36581) was verified by TNHC staff in July 2010 and constitutes a large extension from this species' previously known range [31].

Seven unvouchered historical collections were found, five documenting species not documented by specimen-vouchered records. These include three collections by Tilton (1961; [31]) documenting *Dionda nigrotaeniata*, *Dorosoma cepedianum,* and *Carassius auratus*; Clark Hubbs (1960; unpublished field notes archived at TNHC) documenting *Percina sciera;* and Jordan and Gilbert (1886; [28]) documenting *Ameiurus nebulosus*.

### Species Distribution Models

#### Variable set selection

Despite Maxent's internal model validation tests (AUC and binomial tests) being consistent across models built with the different variable sets and extents, experts unanimously identified as most accurate the same two environmental variable sets, each trained on the Texas political extent, as most closely representing the known ranges of the 16 species modeled for this trial (Table 1). The two variable sets chosen both include the four topographical variables and the supplemental set of categorical variables, and differ by having either all 15 bioclimatic variables or a reduced set of 7 (Table 3).

#### Modeled Community

The FoTX database documents occurrences of 87 freshwater or freshwater-estuarine species as listed in Hubbs et al. ([31]) in the Colorado River basin. Of these, 57 met model construction and validation criteria and were incorporated into the modeled community comparison (Table 4). Of the 30 species not satisfying the study criteria for modeling, nine native and three non-native species have been documented from BCW.

**Figure 2:**
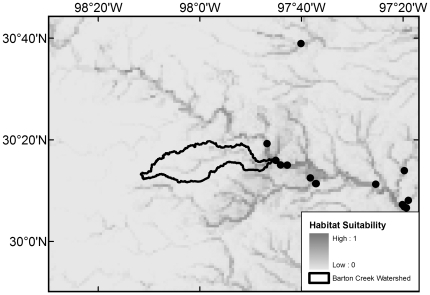
Species Distribution Model for *Opsopoeodus emiliae* (Maximum modeled probability of occurrence in BCW is 0.87). The figure extent is limited to BCW vicinity. The black dots show historical occurrence points in BCW vicinity for this species.

**Figure 3 pone-0025145-g003:**
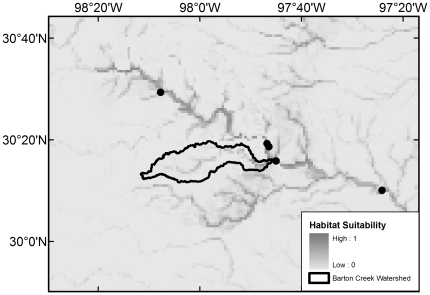
Species Distribution Model for *Notropis shumardi* (Maximum modeled probability of occurrence in BCW is 0.81). The figure extent is limited to BCW vicinity. The black dots show historical occurrence points in BCW vicinity for this species.

**Figure 4 pone-0025145-g004:**
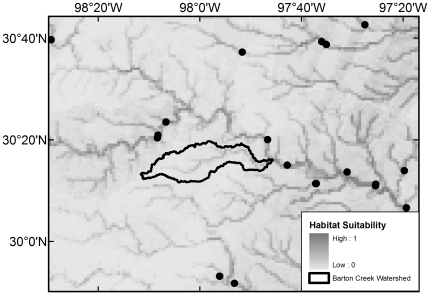
Species Distribution Model for *Lepomis humilis* (Maximum modeled probability of occurrence in BCW is 0.79). The figure extent is limited to BCW vicinity. The black dots show historical occurrence points in BCW vicinity for this species.

**Figure 5 pone-0025145-g005:**
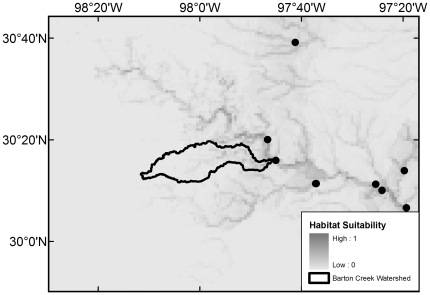
Species Distribution Model for *Hybopsis amnis* (Maximum modeled probability of occurrence in BCW is 0.63). The figure extent is limited to BCW vicinity. The black dots show historical occurrence points in BCW vicinity for this species.

**Figure 6 pone-0025145-g006:**
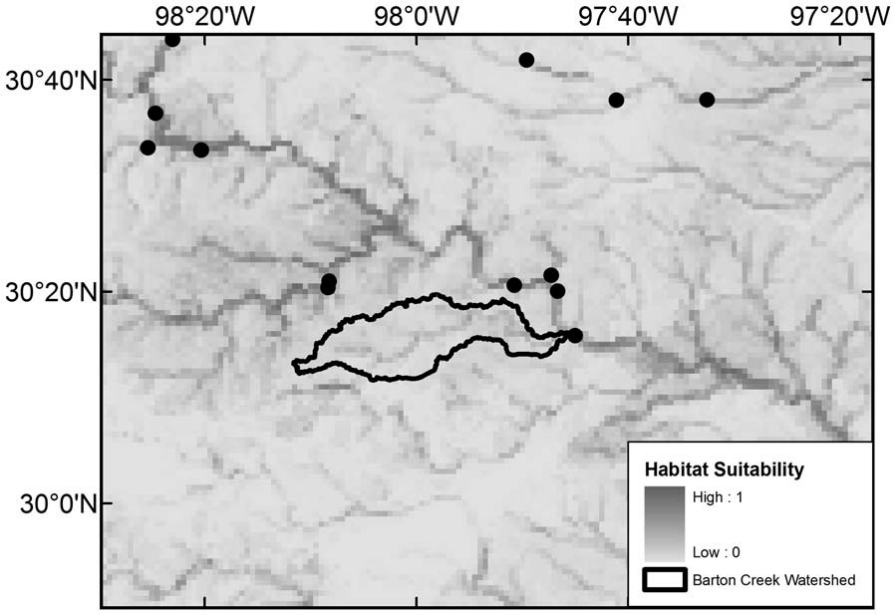
Species Distribution Model for *Percina macrolepida* (Maximum modeled probability of occurrence in BCW is 0.63). The figure extent is limited to BCW vicinity. The black dots show historical occurrence points in BCW vicinity for this species.

**Figure 7 pone-0025145-g007:**
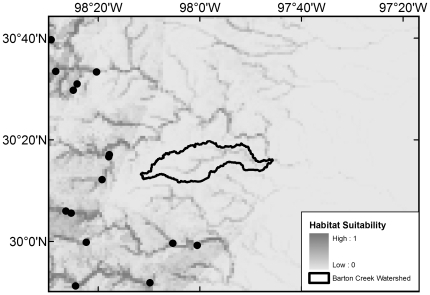
Species Distribution Model for *Notropis stramineus* (Maximum modeled probability of occurrence in BCW is 0.54). The figure extent is limited to BCW vicinity. The black dots show historical occurrence points in BCW vicinity for this species.

**Figure 8:**
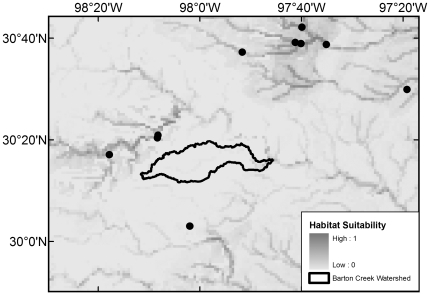
Species Distribution Model for *Notropis buchanani* (Maximum modeled probability of occurrence in BCW is 0.52). The figure extent is limited to BCW vicinity. The black dots show historical occurrence points in BCW vicinity for this species.

All 20 native species with maximum modeled probabilities of occurrence in BCW >87% were collected in the 2008 survey, with the exception of *F. notatus* (max BCW probability 0.95), *P. vigilax* (0.92), and *Etheostoma spectabile* (0.90) (Table 4). The seven species that were most widespread in the 2008 survey are those with the highest modeled probabilities (≥0.94) (Table 4). Of the 34 native species with models that had >50% maximum modeled probability of occurrence in BCW, 16 were not documented in the 2008 survey, 14 were not documented in the 1993 survey, and 10 were not documented in the historical collection data (Table 4). The models indicate occurrence probabilities >0.5 for seven native Colorado River species never recorded in BCW: *Opsopoeodus emiliae* (max BCW probability 0.87; Figure 2), *Notropis shumardi* (0.81; Figure 3), *Lepomis humilis* (0.79; Figure 4), *Hybopsis amnis* (0.63; Figure 5) *Percina macrolepida* (0.63; Figure 6), *Notropis stramineus* (0.54; Figure 7), and *Notropis buchanani* (0.52; Figure 8). Figures 2-8 (respectively) illustrate these seven species' modeled probabilities within the BCW vicinity along with historical occurrence records.

Of the 57 modeled species, only two with a maximum modeled probability of occurrence in BCW <0.5 have ever been documented from the watershed (Table 4). One specimen of *P. carbonaria* (0.30, which also represents the probability at the collection locality) was collected in 2008 below Barton Springs Dam in the lower 500 m of the creek. *Macrhybopsis sp*. (either *M. hyostoma* [0.02] or *M. marconis* [0.15]; both of these probabilities are from the collection locality) was collected by Jordan and Gilbert in 1886 in the lower one km of the creek. This specimen (USNM 36582) was verified by TNHC staff in July 2010, which resulted in a change in identification from *Macrhybopsis aestivalis* to *Macrhybopsis sp*.

## Discussion

The two recent comprehensive surveys of Barton Creek in 1993 and 2008 provide a reliable assessment of the contemporary fish community, documenting 28 & 26 species respectively (24 & 22 native, and four non-native each). All collection records combined, including anecdotal observations, document a total of 45 species (39 native and six non-native). The historical collection data, however, are relatively poor; only one BCW collection was performed before 1950 and later collections prior to 1993 were sparse and done with differing or unknown collection methods.

Modeling techniques used in this analysis provided a critical complement to traditional methodologies of historical reconstruction using only collection data. The modeled community substantiated conclusions drawn from collection records by providing quantitative support for them, and similarly supported inclusion in the historical fauna of seven native species never documented from the watershed. The modeling results also suggest that inclusion of anecdotal-only observations of *D. nigrotaeniata* (max BCW probability 0.56), *D. cepedianum* (0.78), and *P. sciera* (0.87) is likely valid. However, there are limitations in the utilization of an incomplete modeled community for historical reconstruction of an assemblage. Conclusions drawn from the modeled community are likely to under-estimate the true historical community since 18 species known from the Colorado River basin, and thus with potential historical access to the BCW, were omitted from consideration due to limitations imposed by our stringent model construction and validation criteria. For example, the threatened Blue Sucker, *Cycleptus elongatus*, omitted from the modeled community due to poor model quality driven by sparse occurrence data, is known from the Colorado River [28] and there is recent documentation of it spawning immediately below Longhorn dam, 5.5 km downstream of Barton Creek's confluence with the Colorado River, and in nearby tributaries similar in size to BCW [28]. As environmental and biological data become more available through increases in museum database digitization and dissemination, these types of models have much potential to improve in prediction accuracy, permitting inclusion of harder to model species and more precise variable response relationships.

While our analysis does not explicitly address causal mechanisms for deviations from historical condition, it does provide a thorough perspective on the history of the BCW fish community and gives managers a better understanding and ability to infer mechanisms that are influencing community structure. Water development within and downstream of the watershed is likely the largest factor affecting BCW's fish assemblage structure and diversity. Many studies have demonstrated that, compared to larger streams, fish communities of smaller drainages such as Barton Creek are typically less stable and have a higher probability of local extirpations with diversity maintained in large part by repopulation from connected downstream sources [51], [52]. Since construction of Barton Springs dam in 1929, upstream fish movements to re-establish or support fish populations have most likely only been possible during rare and brief high-flow events that allow fish passage around the dam. Additionally, the much larger Longhorn Dam has definitely blocked upstream fish movements on the mainstem Colorado River since 1960. Both dams have thus surely acted for many years to reduce native species diversity in Barton Creek. We hypothesize that these dams may have been major factors in the current (1993 & 2008 surveys) absence from BCW of a number of native regionally ubiquitous and typically abundant species that our models tell us should be found there (e.g., *Cyprinella lutrensis*, *Car. carpio*, *D. cepedianum, N. buchanani, F. notatus*, *P. vigilax*). All of these taxa are know from recent collections to occur within the mainstem Colorado below Longhorn Dam, and/or in other nearby Colorado River tributaries [28].

Biotic interactions also play a role in shaping fish communities as perhaps indicated in BCW by the high modeled habitat suitability of both *E. spectabile* and *E. lepidum,* two ecologically similar taxa [53], [54]. Both have been documented from BCW, however only *E. lepidum,* was found in both recent comprehensive surveys. Additionally, comparison of the two surveys document sharp increases in abundance of non-native species coupled with decreases in abundance of a native keystone herbivore, supporting a hypothesis that biotic interactions between natives and non-natives may be a factor responsible for recent evidence of shifts toward a more invasive-dominated fauna. These invasions are not surprising since models indicated high habitat suitability for five non-native species known from the Colorado River basin; *A. mexicanus* (0.88), *G. geiseri* (0.59), *H. cyanoguttatum* (0.92), *L. auritus* (0.96) and *P. latipinna* (0.97); all but *P. latipinna* and *G. geiseri* now documented to occur in BCW. We were unable to construct valid models for other non-natives known from BCW including *Ctenopharyngodon idella* and *C. auratus.*

This study has implications for stream bioassessment and the use of bioindicators to measure system integrity. The Index of Biotic Integrity (IBI), a multimetric index combining different biotic variables (metrics) correlated with habitat quality [55], is one of the most prevalent techniques for identifying and quantifying systemic aquatic system impacts [55]–[58]. However, IBIs use matrices of region-specific ecological metrics and so are not transferable among regions [23], and they generally do not allow resolution of specific causes of impairment as they aggregate multiple metrics into one score [59]. The technique presented here could be used to develop an analog or complement to IBIs that would measure magnitude of system alteration based on deviation from historical faunal composition. Broadly applied, we propose that this technique has potential to overcome the limitations of IBIs, as stated above, to become a versatile and robust bioassessment tool. As SDMs explicitly incorporate species' responses to explanatory variables across the entire extent utilized in model construction [23], they enable general application of such a bioassessment tool within that extent. For example, as applied in this study we assessed just BCW but modeled the potential species pool for the entire Colorado River basin, producing in the process modeled freshwater fish communities for all drainages within the Colorado River watershed. Additionally, we believe that expanding this analysis, using multiple subbasins as replicates, will allow a robust way to partition and account for broad-scale, often confounding, influences of community diversity (e.g., biotic interactions, fragmentation, altered flows). If our premise that the modeled community constructed in this analysis approximates historical community composition is accepted, congruence between model prediction and contemporary survey results should increase with habitat quality ( = decreasing habitat alterations). We believe that premise to be true and thus, by performing a similar analysis on multiple sets of watersheds that experience clear gradients of particular land or water alterations, we can potentially identify corresponding trends in taxa exclusions or regime shifts and implicate specific causal mechanisms. This application can simultaneously verify the correlation between the congruence of model and survey results when interpreted at different scales, allowing quantification of model accuracy across scales (e.g., locality, reach, subbasin, and watershed) as well as identification of ways that various factors influence community diversity across scales.

In summary, historical data interpreted together with the theoretical historical fauna reconstruction provided by SDMs filled gaps in data coverage and allowed us to draw conclusions that would otherwise not be possible from the disparate historical collections. Contemporary surveys complemented the models by documenting present community composition and recent trends, and allowed a thorough assessment of system status based on the model-derived historical condition. A major benefit of this type of analysis is that it circumvents the rarely satisfied need for excellent biological data on any particular basin or small watershed of interest. By utilizing a regional, and thus substantially larger, biodiversity dataset (in our case primarily natural history museum data) we produced SDM-based habitat suitability estimates for the study region that accounted for broad-scale physiological and zoogeographical constraints. Though each data source used in this analysis has unique values and limitations, it was only by interpreting them together and in the context of the SDMs that we were able to extract a more comprehensive picture of the historical condition of our study area and the factors shaping its fish community. As historical occurrence records increasingly become available through online databases and GIS-generated environmental parameter data improve in spatial and temporal resolution, this technique has much potential as a bioassessment tool, aiding resource managers in setting proper reference baselines.
